# Clinical Work Experiences of Nurses Returning to Work Postpartum: A Systematic Review and Qualitative Meta‐Synthesis

**DOI:** 10.1155/jonm/8747372

**Published:** 2026-04-20

**Authors:** Jing Tian, Yuan Hu, Yuting Yuan, Han Yang, Min Tan, Hong Zheng

**Affiliations:** ^1^ Department of Pediatric Cardiovascular Nursing, West China Second University Hospital, Sichuan University-West China School of Nursing, Sichuan University, Chengdu, China, scu.edu.cn; ^2^ Key Laboratory of Birth Defects and Related Diseases of Women and Children (Sichuan University), Ministry of Education, Chengdu, 610041, Sichuan, China, scu.edu.cn

**Keywords:** meta-synthesis, nurse, postpartum period, qualitative research, return to work

## Abstract

**Background:**

The global nursing shortage underscores the critical need to retain experienced clinicians. The postpartum return to clinical practice represents a vulnerable transition period that can significantly impact nurse retention, yet the nuanced lived experiences of nurses during this reintegration are not well synthesized.

**Aim:**

To systematically review and synthesize qualitative evidence on the clinical work experiences of nurses returning to work after childbirth.

**Design:**

A systematic review and meta‐synthesis.

**Methods:**

A systematic search was conducted in PubMed, Cochrane Library, CINAHL, Web of Science, Embase, China National Knowledge Infrastructure (CNKI), Wanfang Database, Weipu Database and China Biomedical Literature Database (CBM) to identify qualitative studies published between December 1990 and December 2025 that focused on the clinical work experiences of nurses returning to work postpartum. This systematic review follows the meta‐synthesis method guided by ENTREQ and PRISMA and uses the Critical Appraisal Skills Programme (CASP) to assess the quality of included studies. Qualitative findings from the primary studies were integrated and analyzed using thematic synthesis.

**Results:**

A total of 19 studies were included, with 76 main findings extracted, summarized into 9 subthemes, and organized into 3 main themes: The Multi‐Faceted Challenges of Reintegrating into Professional Practice, Enduring Multidimensional Physical and Psychological Strain, and Relying on a Multilayered Support Ecosystem for Successful Transition.

**Conclusion:**

The return to clinical work postpartum is a period of multidimensional strain and identity negotiation. Retention is contingent upon a coherent support ecosystem. Moving beyond broad work–family conflict narratives, this review offers a targeted evidence base supporting essential structural enablers—such as flexible work policies, protected lactation facilities, and tailored reintegration pathways—alongside culturally competent interpersonal support, to promote sustainable reintegration and preserve an experienced nursing workforce.

**Implications for Nursing Management:**

Nurse managers should proactively establish supportive structural policies, including flexible scheduling and dedicated lactation facilities, to support the successful reintegration of postpartum nurses. Developing tailored return‐to‐work orientation pathways and fostering a supportive, empathetic ward culture are critical managerial strategies to mitigate transition‐related stress, enhance job satisfaction, and retain experienced clinical staff amidst the global nursing shortage.

## 1. Introduction

With the World Health Organization projecting a global shortage of 4.5 million nurses by 2030 [1], persistent understaffing and high turnover have made the retention of experienced clinicians a central concern for health systems worldwide [2–4]. Among the various factors influencing nurse retention, the transition back to clinical practice following maternity leave represents a critical yet understudied period. Postpartum nurses constitute a significant subset of the workforce [5], and their successful reintegration is essential for maintaining staffing levels, preserving institutional knowledge, and ensuring high‐quality patient care [6]. However, returning to the physically and emotionally demanding clinical environment while navigating the profound changes of new motherhood presents distinct challenges that may influence job satisfaction, well‐being, and ultimately, career continuity [7, 8].

Existing literature on work–family balance in nursing has often focused broadly on conflicts between generic caregiving roles and work [9, 10] or on quantitative measures of burnout and intent to leave [11, 12]. While these studies highlight overarching tensions, they do not fully capture the nuanced, lived experience of nurses returning postpartum. This period involves specific physiological recovery needs, lactation requirements, intense infant care demands, and a dynamic renegotiation of professional identity [13]—all unfolding within the context of rigid shift work, high‐acuity patient loads, and often inflexible institutional policies [14]. A deeper qualitative understanding of this experience is crucial. Without it, healthcare leaders and policymakers lack the evidence required to develop targeted, effective support structures that can support a sustainable transition, thereby promoting the well‐being of these nurses and safeguarding their valuable place within the profession. However, to date, no synthesis has comprehensively integrated qualitative evidence regarding how postpartum nurses specifically experience and navigate the complex process of reintegration into clinical practice.

Therefore, this study aims to synthesize qualitative evidence on the clinical work experiences of nurses returning to work after childbirth. Through a systematic review and qualitative meta‐synthesis, we seek to integrate findings from relevant studies to construct a comprehensive, interpretative understanding of this phenomenon. By elucidating the key themes, challenges, facilitators, and coping mechanisms that characterize this transition, this review aims to provide a robust evidence base to inform the development of supportive workplace interventions, compassionate policies, and further research, ultimately contributing to a more resilient and sustainable nursing workforce.

## 2. Methods

### 2.1. Research Design

This study employed the methodology of a systematic review and qualitative meta‐synthesis. The inclusion and exclusion criteria were developed based on the PICo framework from the Joanna Briggs Institute (JBI) [15]. This framework structured the criteria around Population (“P”), the phenomenon of Interest (“I”), and Context (“Co”). Furthermore, to ensure transparent reporting, this review adhered to the Preferred Reporting Items for Systematic Reviews and Meta‐Analyses (PRISMA) statement [16] and the ENTREQ guidelines [17]. Detailed checklists are provided in supporting B and supporting C. For data synthesis, a thematic analysis approach was applied [18]. This study was registered in the PROSPERO International Prospective Register of Systematic Reviews (registration ID: CRD420251244220).

### 2.2. Search Strategy

A systematic search was conducted across multiple electronic databases, including PubMed, Cochrane Library, CINAHL, Web of Science, Embase, CNKI, Wanfang Database, Weipu Database, and CBM to identify qualitative studies published between December 1990 and December 2025 that focused on the clinical work experiences of nurses returning to work postpartum. The search strategy combined subject headings (e.g., MeSH in PubMed) and free‐text terms tailored to the specific features of each database. Key search terms included “nurs∗”, “return to work”, “postpartum return”, “back to work”, “maternity leave”, “parental leave”, “experience”, “feeling”, “interview”, “attitude”, “perception”, “view”, “perspective∗”, “qualitative research”, “qualitative study”, “qualitative”, “content analysis”, “discourse analysis”, “ethnography”, “focus group”, “grounded theory”, “narrative”, “phenomenology”, and “thematic analysis”. To ensure comprehensive coverage, manual citation tracking of the reference lists of included studies and relevant reviews was also performed. The full search strategy is provided in supporting A.

### 2.3. Inclusion and/or Exclusion Criteria

Inclusion criteria: ① Participants (P): Registered nurses (regardless of clinical department or professional title) who have experienced returning to work after childbirth (i.e., returning to work after maternity or parental leave). ② Interest of Phenomena (I): Focus on exploring the clinical work experiences, feelings, perceptions, challenges, facilitating factors, and coping strategies of postpartum nurses returning to work. ③ Context (Co): Occurring in the clinical environment of any healthcare institution (e.g., hospital wards, emergency departments, community clinics, etc.). ④ Study types (S): Qualitative study designs, including but not limited to phenomenology, grounded theory, ethnography, case studies, descriptive qualitative research, etc. Language: Chinese or English.

Exclusion criteria: Studies where participants are solely non‐nurse healthcare professionals, studies where data specific to nurses cannot be disaggregated; studies primarily focusing on experiences during maternity leave, solely physiological breastfeeding issues, parenting stress, or nonclinical experiences after returning to work; studies conducted in nonclinical settings; quantitative studies; mixed‐methods studies (if the qualitative data cannot be obtained and analyzed separately); systematic reviews; literature reviews; commentaries, editorials, conference abstracts, or studies without full text available; studies published in languages other than Chinese or English; studies for which the full text cannot be obtained; duplicate publications.

### 2.4. Study Selection and Data Extraction

The retrieved literature was managed within EndNote 20 for the identification and removal of duplicates. The screening process was conducted independently by two reviewers trained in evidence‐based research methods, who assessed the titles, abstracts, and subsequently the full texts of potentially eligible studies against the predefined inclusion and exclusion criteria. Any discrepancies arising during the screening or data extraction phases were resolved through discussion or, when necessary, adjudication by a third reviewer. Following the initial title/abstract screening to exclude irrelevant records, the remaining articles underwent a full‐text review for final eligibility determination. Pertinent data, including author(s), country of origin, study population, methods of data collection and analysis, as well as key findings or themes, were systematically extracted from the included studies.

### 2.5. Quality Appraisal

The methodological quality of the included studies was appraised using the Critical Appraisal Skills Programme (CASP) checklist for qualitative research [19]. This appraisal aimed to evaluate the rigor, transparency, and applicability of each study to support the credibility of the synthesis findings. The CASP checklist comprises 10 core items, preceded by two initial screening questions assessing the clarity of research aims and appropriateness of the qualitative methodology. The remaining items systematically evaluate key domains, including study design, recruitment strategy, data collection, reflexivity in researcher–participant relationships, ethical considerations, analytical rigor, clarity of findings, and research value.

Two reviewers independently assessed all studies. Any discrepancies in their evaluations were resolved through consensus discussion and, when necessary, consultation with a third researcher. The results of the quality assessment are summarized in Table 1, detailing the performance of each study across the evaluated domains.

**TABLE 1:** Quality assessment of included studies.

Included study	Q1	Q2	Q3	Q4	Q5	Q6	Q7	Q8	Q9	Q10
Chen et al., [20]	Y	Y	Y	Y	Y	N	Y	Y	Y	Y
Costantini et al., [21]	Y	Y	Y	Y	Y	C	Y	Y	Y	Y
Hill et al., [22]	Y	Y	Y	Y	Y	Y	Y	Y	Y	Y
Khalil & Davies, [23]	Y	Y	Y	C	Y	C	Y	C	Y	Y
Li, [24]	Y	Y	Y	Y	Y	C	Y	Y	Y	Y
Li et al., [14]	Y	Y	Y	Y	Y	Y	Y	Y	Y	Y
Liu et al., [25]	Y	Y	Y	Y	Y	N	Y	Y	Y	Y
Riaz & Condon, [26]	Y	Y	Y	Y	Y	N	Y	Y	Y	Y
Tseng et al., [27]	Y	Y	Y	Y	Y	C	Y	Y	Y	Y
Wan et al., [28]	Y	Y	Y	Y	Y	C	Y	Y	Y	Y
Zhou T., et al. [29]	Y	Y	Y	Y	Y	Y	Y	Y	Y	Y
Guo et al. [30], China	Y	Y	Y	Y	Y	C	Y	Y	Y	Y
Li et al. [31], China	Y	Y	Y	Y	Y	C	Y	Y	Y	Y
Huang et al. [32], China	Y	Y	Y	Y	Y	N	Y	Y	Y	Y
He et al. [33], China	Y	Y	Y	Y	Y	N	Y	Y	Y	Y
Xiang et al. [34], China	Y	Y	Y	Y	Y	N	Y	Y	Y	Y
Yang et al. [35], China	Y	Y	Y	Y	Y	N	Y	Y	Y	Y
Cheng et al. [36], China	Y	Y	Y	Y	Y	N	Y	Y	Y	Y
Xie et al. [37], China	Y	Y	Y	Y	Y	N	Y	Y	Y	Y

*Note:* Y = yes; C = cannot tell; N = no. The Critical Appraisal Skills Program Questions: (1) Was there a clear statement of the aims of the research? (2) Is a qualitative methodology appropriate? (3) Was the research design appropriate to address the aims of the research? (4) Was the recruitment strategy appropriate for the aims of the research? (5) Was the data collected in a way that addressed the research issue? (6) Has the relationship between the researcher and participants been adequately considered? (7) Have ethical issues been taken into consideration? (8) Was the data analysis sufficiently rigorous? (9) Is there a clear statement of the findings? (10) How valuable is the research/will the results help locally?

### 2.6. Data Synthesis

The data analysis and synthesis in this review were conducted following the three‐stage thematic synthesis method outlined by Thomas and Harden [18]. This structured approach comprises (1) coding of textual data, (2) development of descriptive themes, and (3) generation of analytical themes [38].

Using Microsoft Excel, two researchers independently performed line‐by‐line coding of participant quotes and author interpretations. To ensure coding consistency, a preliminary coding framework was established using a small subset of studies before analyzing the remaining data. For instance, initial codes such as “lack of private space” and “insufficient break time” were grouped into descriptive themes reflecting logistical barriers. Subsequently, a third researcher reviewed the initial codes and developed the themes. Through iterative team discussions, descriptive concepts were refined into broader analytical themes—for example, synthesizing “persistent fatigue” and “postpartum physical recovery” into the overarching theme “Enduring Multidimensional Physical and Psychological Strain.”

Any discrepancies in coding were resolved through collaborative discussion, with a senior researcher adjudicating unresolved disagreements to ensure interpretive rigor. Finally, the entire research team reviewed the complete data analysis process. Meta‐themes were developed through the synthesis of analytical themes to capture the work experiences of nurses returning to work postpartum, as presented in the Results section.

### 2.7. Confidence Analysis

Confidence in the key review findings was evaluated using the GRADE‐CERQual approach [39]. This methodology assesses confidence based on four components: (1) methodological limitations of the contributing studies, (2) coherence of the finding, (3) adequacy of the data supporting the finding, and (4) relevance of the contributing studies to the review question. In strict accordance with GRADE‐CERQual guidelines, each component was systematically appraised for every finding. An overall judgment of confidence for each finding was then made, categorizing it as high, moderate, low, or very low.

## 3. Results

### 3.1. Search Results

**FIGURE 1:**
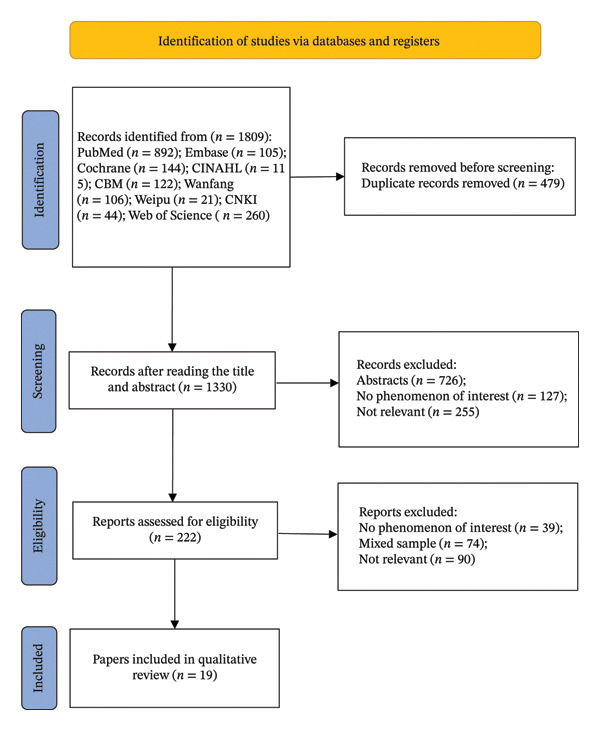
PRISMA flowchart for literature search.

In the initial retrieval, a total of 1809 articles were identified through database searches. After importing the records into EndNote, 479 duplicate records were removed. Subsequently, the titles and abstracts of 1330 articles were screened, of which 1108 were excluded based on the eligibility criteria. Following this, 222 full‐text articles were assessed for eligibility, and 202 were excluded. Finally, 19 full‐text articles met the inclusion and exclusion criteria and were included in the final synthesis. The selection flow diagram is presented in Figure 1.

### 3.2. Description of Included Studies

Among the 19 included studies, the sample sizes ranged from 5 to 20 participants, yielding a total sample of 231 individuals. The studies were conducted in the following countries: China (*n* = 15), Italy (*n* = 1), USA (*n* = 1), UK (*n* = 1), and Pakistan (*n* = 1). Methodologies employed across the studies included phenomenological qualitative research (*n* = 13), descriptive qualitative research (*n* = 4), qualitative interpretative description (*n* = 1), and grounded theory (*n* = 1). Regarding data collection methods, 18 studies utilized semistructured interviews, while one study employed a combination of one‐on‐one interviews and focus groups. For details, please refer to Table 2.

**TABLE 2:** Characteristics of the studies included.

Authors, year, country	Methodology	Data collection	Participants	Data analysis	Key themes
Chen et al., 2024, China	Qualitative descriptive design using phenomenological approach	Face‐to‐face semistructured in‐depth interviews (35 interviews)	20 inexperienced lactating nurses (≤ 5 years of experience)	Colaizzi’s method using NVivo12	1. Challenges faced during the breast feeding period (Health effects, Lack of sleep, Conflict between work and breast feeding, Harassment fear). 2. Conflicting professional and family roles (Job‐ability mismatch, Role confusion). 3. Out of balance (Workplace conflicts, Occupational exposure panic, Relative pressure). 4. Coping strategies (Self‐adjustment, Effective communication, Facing up to the dilemma).

Costantini et al., 2022, Italy	Qualitative interpretative description	Face‐to‐face semistructured interviews (12 interviews, 50 min each)	12 nurses aged 31–43, returned to work 2–8 months prior	Qualitative content analysis	1. Children nurturing (Knowing the baby is in good hands, Mother–child separation). 2. Family and work (Two worlds that enrich each other, The challenge of combining the two worlds). 3. Loss and gains (Experiences as a novice, Expertise gains). 4. Handling the return to work.

Hill et al., 2023, USA	Qualitative descriptive study	One‐on‐one interviews and focus groups, 30–60 min each	19 emergency nurses returned from parental leave within 6 months to 2 years	Braun and Clarke’s 6‐phase thematic analysis	1. Work engagement (Lack of communication, Perceived engagement expectations, Actual engagement). 2. Lactation (The act of pumping, Lactation breaks, Lactation rooms). 3. Childcare.

Khalil & Davies, 2000, UK	Qualitative grounded theory approach	Semistructured interviews (5 interviews, taped and transcribed)	5 first‐time mothers	Grounded theory–based content analysis	1. Continuing career pathways 2. Coping mechanisms 3. Self‐perception 4. Division of labor 5. Relationships with partners

Li, 2024, China	Empirical phenomenological approach	Semistructured video interviews (15 interviews, 43–76 min), purposive and snowball sampling	15 lactating nurses who returned to work within 44 days postpartum, all had COVID‐19 infection history	Colaizzi’s seven‐step method	1. Preparation for return to work Feed preparation, Self‐directed learning, Choice of caregiver 2. Experiences of return to work Breastfeeding challenges, Physical challenges, Work adaptation, Emotional burden 3. Experiences of infection Preventive behaviors before infection, Responses to infection, Post‐recovery reflections

Li et al., 2025, China	Descriptive phenomenological study	Individual in‐depth, semistructured interviews (50–75 min), conducted in Mandarin Chinese	17 neonatal nurses who returned to work within 1–12 months postpartum; purposive sampling with maximum variation	Colaizzi’s seven‐step phenomenological analysis using NVivo	1. Establishment of maternal identity and life reorientation 2. Multifaceted adaptation challenges (anxiety, physical/psychological impacts, skill readjustment, breastfeeding‐work conflict) 3. Maternal identity enhances empathy and transforms care behaviors 4. Ecosystem factors (support or barriers) influence transition

Liu et al., 2024, China	Descriptive phenomenological approach	Semistructured interviews (30–60 min), conducted in quiet settings, audio‐recorded and transcribed	12 postnatal nurses; convenience sampling; all had 2–3 children and returned to work within 3 months	Colaizzi’s 7‐step method	1. Postnatal physical decline (fatigue, pelvic floor disorder, memory decline, sleep disturbance) 2. Postnatal psychological maladjustment (work–family conflict, breastfeeding conflict, role maladjustment) 3. Lack of clear career planning

Cheng et al. 2025, China	Phenomenological Research	Semistructured in‐depth interview	7 returning nurses who insisted on breastfeeding	Colaizzi’s Phenomenological Seven‐Step Analysis Method	4 themes emerged: significant pressure to maintain breastfeeding; decreased physical capacity to cope with workload; conflict between work and family roles; low job satisfaction and poor coping confidence.

Riaz & Condon, 2019, Pakistan	Qualitative descriptive approach	In‐depth semistructured interviews in Urdu, audio‐recorded and transcribed	7 purposively sampled nurses breastfeeding at the time of return to work	Iterative thematic analysis (Pollio, Henlay & Thompson approach)	1. A child’s right to breastfeed 2. Conflict with institutional power (rigid policies, short maternity leave, no childcare) 3. Importance of family support (especially fathers bringing babies to hospital for feeding)

Tseng et al., 2023, China	Qualitative descriptive approach	Face‐to‐face semistructured interviews (45–60 min), audio‐recorded and transcribed	13 female nurses who had applied for parental leave; purposive sampling	Content analysis using Graneheim and Lundman’s method	1. Considerations for taking parental leave (childcare, finances, desire to raise child) 2. Support received (family, superiors, wage subsidies) 3. Life during leave (joy, isolation, disconnection from society) 4. Concerns about returning (skill loss, child adjustment) 5. Preparations for return (childcare, relearning, routine adjustment)

Wan et al., 2024, China	Qualitative descriptive design	Face‐to‐face semistructured interviews (30–60 min), conducted in nurses’ lounge during non‐working hours	8 breastfeeding nurses within 6 months of return; purposive sampling	Braun and Clarke’s thematic analysis using NVivo 12	1. Changes in nurses (emotional, physical, and work‐related) 2. Needs for an improving work environment (supportive space, nurse shortage issues) 3. Support for breastfeeding nurses (from coworkers, managers, organization, and self)

Zhou et al. 2024, China	Descriptive phenomenological approach	Individual semistructured face‐to‐face interviews (30–45 min) in a private hospital setting; audio‐recorded with field notes; March–May 2023	16 registered nurses who had returned to direct patient‐care roles within 1 year after maternity leave; purposive and maximum‐variation sampling (variation by age, education, title, years of experience, unit, number of deliveries, time since return)	Colaizzi’s 7‐step descriptive phenomenological analysis using NVivo 12; credibility via pilot interviews, member checking, reflexive meetings and audit trail	1. “Changes and challenges of multiple roles” (physical & mental exhaustion, breastfeeding & separation anxiety, disagreements within the family, disconnection from work, changes in career planning) 2. “Self‐coping and social support” (positive self‐management, spousal coordination, family support, peer support) 3. “Further needs after returning to work” (flexible working arrangements, proactive care from supervisors and colleagues, equitable policy for experiencing multiple deliveries, well‐established mother–baby facilities)

Huang et al. 2018, China	Phenomenological Research	Semistructured in‐depth interview	6 postpartum returning nurses with a second child	Colaizzi’s seven‐step method	5 work experiences emerged: concerns about child‐rearing; difficulties in adaptation and self‐psychological adjustment; positive emotional experiences and expressions; intense physical and mental exhaustion; and work–family conflict. 3 support needs were identified: scheduling support; the desire for an adaptation period; and colleague support.

Li et al. 2017, China	Phenomenological Research	Face‐to‐face, semistructured interviews	14 postpartum returning nurses from the operating room	Colaizzi’s seven‐step method	4 themes were identified: adverse subjective feelings (primarily including fatigue, pain, and discomfort); psychological burden (mainly manifested as worry, irritability, fear, and guilt); potential safety hazards at work (characterized by a lack of work continuity and difficulty concentrating); and the need for team support.

He et al. 2020, China	Phenomenological Research	Face‐to‐face, semistructured interviews	14 postpartum returning nurses	Colaizzi’s seven‐step method	4 themes were identified: work–family conflict; lack of return‐to‐work training; low self‐efficacy; and insufficient adaptability upon returning to work.

Xiang et al. 2023, China	Phenomenological Research	Face‐to‐face, semistructured interviews	8 lactating postpartum returning nurses from the pediatric ward	Colaizzi’s seven‐step method	4 themes emerged: decreased physical capacity to cope with workload; imbalance between work responsibilities and family roles; inadequate organizational support systems for returning nurses; and low self‐confidence in coping with return‐to‐work challenges.

Yang et al. 2024, China	Phenomenological Research	Semistructured in‐depth interview	15 postpartum returning nurses from the pediatric emergency department	Colaizzi’s 7‐step analysis method, using Nvivo 7.0	4 themes were identified: difficulties in life and work; psychological changes after returning to work; role conflict; and the desire for support from both the workplace and family.

Guo et al. 2024, China	Interpretive Phenomenological Research	Semistructured in‐depth interview	12 postpartum returning nurses	Classification analysis method	4 themes emerged: fear of returning to work; declining physical condition; conflict between family and work; maladaptation to environmental changes.

Xie et al. 2026, China	Phenomenological Research	Semistructured in‐depth interview	11 postpartum returning nurses from the oncology department	Colaizzi’s Phenomenological Seven‐Step Analysis Method	4 themes were identified: declining physical condition (fatigue, pain); psychological stress (anxiety, tension, fear, guilt); decreased work quality; and the expectation of support from the workplace and family.

### 3.3. Confidence in the Findings

The GRADE‐CERQual approach [39] was used to assess confidence in each of the nine subthemes: two were rated as moderate confidence and seven as high confidence. For details, see Table 3 and supporting D.

**TABLE 3:** Themes, sub‐themes, and CERQual confidence ratings.

Theme	Subtheme	Brief description	CERQual confidence
Theme 1: The Multi‐Faceted Challenges of Reintegrating into Professional Practice	1.1. Physiological and Infrastructural Barriers	Conflict between breastfeeding needs and rigid workplace infrastructure (e.g., lack of private lactation rooms/time), leading to physical pain and reduced milk volume.	High
	1.2. Professional and Maternal Identity Reconciliation	Cognitive dissonance between “nurse” and “mother” roles; experiences range from role confusion/guilt to positive professional growth (increased empathy).	High
	1.3. Maternal Separation and Childcare Logistics	Stress arising from inflexible rosters incompatible with newborn needs, intense separation anxiety, and a lack of reliable childcare options.	High

Theme 2: Enduring Multidimensional Physical and Psychological Strain	2.1. Physical Depletion and Health Concerns	Profound exhaustion, sleep deprivation, and postpartum recovery issues (e.g., pain, incontinence); fear of occupational hazards/infections affecting the infant.	High
	2.2. Emotional and Cognitive Burden	Pervasive guilt and anxiety; cognitive challenges (e.g., memory lapses, concentration decline); pressure to maintain a “Supernurse” image despite internal distress.	High
	2.3. Professional Reintegration Anxiety	Fear of skill decay and feeling de‐professionalized; stress associated with relearning protocols/technologies; uncertainty regarding long‐term career trajectory.	Moderate

Theme 3: Relying on a Multilayered Support Ecosystem for Successful Transition	3.1. Interpersonal Support as a Critical Buffer	Crucial support received from family (childcare/housework), colleagues (emotional solidarity/shift cover), and managers (schedule flexibility/advocacy).	High
	3.2. Organizational and Structural Enablers	The need for systemic changes, including flexible work arrangements, onsite facilities (lactation rooms/childcare), extended leave policies, and formal re‐entry training.	Moderate
	3.3. Personal Agency and Coping Strategies	Active utilization of cognitive self‐management (reframing), preparation (refreshing skills/stockpiling milk), and proactive communication to negotiate needs.	High

### 3.4. Main Findings of the Meta‐Synthesis

Following the three‐stage thematic synthesis approach outlined by Thomas and Harden [18], the analysis of 19 qualitative studies yielded three analytical themes and nine descriptive subthemes. The synthesis moved from line‐by‐line coding of primary study findings, through the development of descriptive themes, to the generation of these overarching analytical themes that help interpret and contextualize the experiences across the dataset. A summary of the identified themes, subthemes, and their corresponding GRADE‐CERQual confidence ratings is presented in Table 3.

#### 3.4.1. Theme 1: The Multifaceted Challenges of Reintegrating Into Professional Practice

##### 3.4.1.1. Subtheme 1.1. Physiological and Infrastructural Barriers

This subtheme focuses specifically on the physical and environmental conflicts inherent in the workplace. It highlights the friction between the biological necessity of breastfeeding and the rigid infrastructure of the clinical environment. Nurses frequently reported inadequate institutional support, citing a lack of dedicated, private, and hygienic lactation rooms, which often led them to express milk in unsuitable spaces such as storage closets or bathrooms [22, 26, 29]. This was compounded by the insufficiency of protected break time, making adherence to the pumping schedules required for physiological milk maintenance nearly impossible [24]. These logistical failures often contributed to physical and psychological consequences, including decreased milk volume associated with stress and fatigue [20, 24]. Chinese qualitative studies echo these findings, emphasizing the intense physical pressure of breastfeeding maintenance. Research on lactating nurses highlights how the demanding clinical duties and insufficient institutional support exacerbate the challenge of adhering to breastfeeding schedules [36, 37].

“We almost always pump in the nurses’ duty room, but it’s inconvenient with a lot of colleagues, and there’s no way to ensure privacy. There are a lot of female nurses, and I think there is a need for a private space in the department to be used as a mother‐and‐baby room.”[29].

“I used to pump out excess milk and store it in the fridge.”[24].

“I started to adapt my baby to milk powder.”[24].

##### 3.4.1.2. Subtheme 1.2. Professional and Maternal Identity Reconciliation

Distinct from logistical or physical challenges, this subtheme addresses the cognitive and emotional labor of identity integration. It describes the internal process of resolving the dissonance between the “nurse” and the “mother.” Many nurses experienced role confusion, feeling torn between the ethos of uninterrupted patient care and the compelling needs of their infant, which could foster a sense of professional disconnection and imbalance [20, 21, 25, 29]. However, for others, particularly those in specialty areas like neonatal care, motherhood prompted a positive transformation of professional self. They reported a deepened sense of empathy, a recalibrated perspective on family‐centered care, and a more holistic approach to their practice, summarized by the notion that maternal experience made them “better nurses” [14]. These experiences of identity conflict and transformation are also prevalent in Chinese contexts. Studies report that while nurses grapple with role confusion and guilt, many also describe positive growth and a more holistic approach to patient care, particularly in emotionally demanding units [25, 32].

“When drawing blood from a baby and he′s wailing, my heart aches as if I′m being stabbed. I literally break out in a sweat…It feels like their pain is my own. I find myself staying longer to comfort them.”[14].

“Now I actively spend extra time reassuring anxious mothers, because I truly understand their fears.”[14].

##### 3.4.1.3. Subtheme 1.3. Maternal Separation and Childcare Logistics

This subtheme focuses on the relational and operational interface between home and hospital. It addresses the tangible strain of scheduling and the emotional weight of physical separation. A primary stressor was the incompatibility of inflexible nursing rosters with the relentless needs of a newborn, contributing to exhaustion and “time‐based” conflict [25]. This logistical friction was closely tied to maternal separation anxiety; nurses described profound guilt and distress when leaving their infants, with concerns for the child’s well‐being intruding on their professional focus [21, 24, 29]. Consequently, securing reliable childcare was a critical barrier to successful return, with a lack of accessible options often provoking significant anxiety [24, 26, 27]. Chinese research consistently underscores these specific operational challenges. Themes such as “worries about child rearing” and “imbalance between work and family roles” are recurrent, highlighting the universal struggle to manage childcare responsibilities without adequate support [30, 33, 34].

“When I initially returned to work, the separation anxiety as a mother was even greater than that of the baby. I felt that he might not eat or sleep well if I was not with him, so I would sometimes get distracted when I was at work.”[29].

“My family all encouraged me to go to work and provided strong support…this made me feel very secure about working.”[14].

“I often watched my baby through home monitoring systems.”[24].

#### 3.4.2. Theme 2: Enduring Multidimensional Physical and Psychological Strain

##### 3.4.2.1. Subtheme 2.1. Physical Depletion and Health Concerns

The return to work was marked by a profound state of bodily exhaustion. Persistent fatigue and sleep disruption were universal, arising from the combination of newborn care, potential postpartum sleep disturbances, and the physical demands of clinical shifts [20, 25]. Concurrently, nurses were managing ongoing postpartum physical recovery, reporting issues such as back pain, pelvic floor dysfunction, perineal pain, and swollen feet, which were often aggravated by prolonged standing and patient‐handling duties [24, 25]. This vulnerability was heightened by a heightened perception of occupational risk. Nurses expressed intense fear of exposure to infectious agents (particularly salient post‐COVID‐19), physical injury from lifting, and workplace violence, now framed as a significant risk not only to themselves but also to their infant’s health [20, 24]. This physical vulnerability appears to be universal across healthcare settings, yet it is particularly acute in high‐intensity units. Qualitative evidence from China indicates that nurses returning to operating rooms and oncology departments perceive a distinct decline in physical function that can compromise their ability to cope with standard clinical workloads. In these contexts, the physical toll is not merely a discomfort but a functional barrier to professional performance [31, 37].

“I sleep for 4 or 5 h every day … I feel a lack of sleep” [24].

“I experienced postpartum urinary incontinence when I laughed out loud or coughed. I felt the involuntary discharge of urine, which made me uncomfortable, especially in the workplace.”[25].

“After delivery, I was unable to hold as much urine in my bladder. I was faced with frequent urination, which meant that I did not dare drink too much water at work.”[25].

“We are in an infectious disease unit where our patients are afflicted with serious contagious diseases. I am genuinely afraid of infecting my child, so I make a point to cleanse myself thoroughly twice at the hospital before having contact with my child at home. Sometimes, after being in close proximity to severely infected patients in the unit, I am terrified to go home. I constantly feel unclean.”[20].

##### 3.4.2.2. Subtheme 2.2. Emotional and Cognitive Burden

Distinct from specific clinical insecurities, this subtheme captures the generalized psychosocial distress and physiologically associated cognitive fatigue linked to the dual demands of early motherhood and nursing. The physical strain was closely intertwined with significant psychological distress. A pervasive sense of guilt and internal conflict was reported: guilt toward the infant for being absent, guilt toward colleagues for perceived reduced capacity or needing accommodations, and guilt toward partners for an unequal division of domestic labor [23, 24]. This coexisted with pronounced generalized anxiety, stress, and feeling overwhelmed by the cumulative demands, sometimes manifesting as panic related to occupational exposure or the work–life balancing process [14, 29]. Some nurses also noted subjective cognitive changes associated with the postpartum physiological state, such as memory lapses or a perceived decline in concentration, which compounded their overall psychological vulnerability [25]. In parallel, Chinese literature frames this cognitive burden as a potential patient safety issue. Nurses in procedural areas, such as operating rooms, report that emotional irritability and difficulty concentrating—stemming from role conflict—can contribute to a “lack of continuity” in work. This suggests that the emotional burden of returning to work extends beyond individual well‐being to impact clinical safety culture [31, 36].

“I felt guilty about my co‐workers … they had to take more work.”[24].

“I was definitely anxious because I hadn′t been working for so long…I might not do well in my job… and my memory felt super bad after giving birth.”[14].

“My memory worsened after the delivery, it was not an exaggeration to say that on my first day back at work, I did not know how to use an indwelling needle.”[25].

##### 3.4.2.3. Subtheme 2.3. Professional Return‐to‐Work Anxiety

While Subtheme 2.2 details internal emotional distress and biological cognitive fatigue, this subtheme focuses strictly on external, competence‐driven anxieties rooted in the clinical environment. A major fear was perceived skill decay and feeling de‐professionalized. Nurses worried that their clinical knowledge and technical skills had eroded during their leave, leaving them feeling like a “novice” or “newcomer” again, which undermined their professional confidence [21, 27]. This was coupled with the need for rapid adaptation to workplace changes, including new protocols, technology, and team dynamics, requiring a period of intensive relearning [24, 27]. Looking forward, some nurses experienced uncertainty regarding their overarching career trajectory. The overwhelming nature of the return period sometimes resulted in a lack of clear career planning, with some questioning their long‐term future in the profession [25]. This phenomenon is conceptualized in recent Chinese studies as an “adaptation disorder” frequently accompanied by low self‐efficacy. Research focusing on nurses returning to specialized pediatric emergency departments and wards highlights that the fear of “maladaptation to environmental changes” is a predominant stressor, reinforcing the need for targeted professional re‐socialization [25, 33].

“I was worried coming back. Work content may be different. Many procedures or details at work might have changed. I have been disconnected with work for some time, I have doubts that I may not be able to resume my previous work and become a problem for others!”[27].

“I had to relearn techniques … prone ventilation, high‐flow oxygen therapy, and so on.”[24].

“After giving birth, I had no time for myself. I found that some of my friends were improving themselves by pursing a higher degree or learning new clinical skills. By contrast, I did not know what I would do next.”[25].

#### 3.4.3. Theme 3: Relying on a Multilayered Support Ecosystem for Successful Return

##### 3.4.3.1. Subtheme 3.1. Interpersonal Support as a Critical Buffer

Support from close networks was the most frequently cited facilitator. Spousal and family support was foundational, involving practical help with childcare and housework, as well as emotional encouragement and affirmation. In some contexts, family members brought the infant to the hospital for feeding [26, 29]. Peer and collegial support was invaluable for daily functioning. Empathetic colleagues provided practical cover during lactation breaks, shared similar experiences reducing feelings of isolation, and offered emotional solidarity [28, 29]. Managerial and supervisory support was a pivotal factor. Supportive managers who offered schedule flexibility, checked in proactively, and advocated for the nurse’s needs within the unit created a psychologically safe environment that significantly eased the transition [28, 29]. The criticality of this support ecosystem is further magnified in the context of multiparous nurses. Studies on nurses returning after a second child in China emphasize that strong “team support” and familial backing are key buffers against the intensified role conflict associated with expanding family structures [32, 37].

“When my baby was sick, my husband told me to go to work without worrying and he would stay at home to take care of the baby. He stayed up all night monitoring the baby’s temperature.”[29].

“Whenever I have a problem taking care of my children, my coworkers will discuss together and pass on their experience.”[28].

“The manager will arrange for us to work in the triage positions for a while so that we could get used to the environment and our strength could gradually recover. Although I am quite busy now, I was not as tired as others.”[28].

##### 3.4.3.2. Subtheme 3.2. Organizational and Structural Enablers

Nurses identified key systemic factors that would enable a more sustainable return. There was a strong consensus on the need for flexible work arrangements, such as part‐time options, phased returns, or adjusted shifts, to allow for a gradual readjustment [29]. The provision of adequate mother–infant facilities was an essential practical need, encompassing not just lactation rooms but also on‐site or subsidized childcare [22, 29]. Furthermore, nurses critiqued existing parental leave policies as often being too short and financially inadequate, calling for extensions and equitable policies that account for multiple childbirths [25, 26, 29]. Building on these structural recommendations, Chinese nurses explicitly identify the absence of formal “re‐entry training” as a key institutional failure. There is a specific call for structured “adaptation periods” and skill‐refreshment programs to bridge the gap between leave and clinical practice, particularly for those in high‐acuity settings [33, 34].

“If some nurses don’t want to be bothered by family chores and want to return to work early, I think the nursing managers should support them and not deduct their maternity allowance. I hope that the managers could offer more flexibility in choosing the time to return to work.” [29].

“I hope that when I give birth to my second child, I will not have to work the night shift until the baby is one year old. Because of the lack of staff, some mothers are required to work the night shift just one month after returning to work.” [29].

“In the long run, it is difficult to find a suitable nanny, especially for dual‐career or multi‐child families like us. If the hospital could organize a childcare centre, we would all be willing to pay for it and would feel more relaxed.”[29].

“I didn’t get my authorized annual leave after my maternity leave. Although I wanted to take it for my baby but it was said that due to shortage of staff it was not possible… This hospital had problems much bigger than ours.”[26].

##### 3.4.3.3. Subtheme 3.3. Personal Agency and Coping Strategies

Alongside external support, nurses actively employed personal strategies. Cognitive and emotional self‐management was common, including positive self‐talk, consciously lowering self‐expectations, and engaging in activities to reduce stress [20, 29]. Many engaged in proactive preparatory work before returning, such as refreshing clinical knowledge online, establishing structured childcare routines, and stockpiling breast milk [24, 27]. A key behavioral strategy was seeking and initiating effective communication with supervisors, partners, and family to explicitly articulate their needs and negotiate solutions [20, 28]. Complementing these behavioral strategies, findings from China highlight the importance of “positive emotional expression” as a resilience mechanism. Despite facing high levels of burnout, nurses actively utilize psychological regulation to navigate the “difficulty of self‐adaptation,” suggesting that internal cognitive reframing is as vital as external negotiation [32, 36].

“I found that exercise is very important, especially outdoor activities. Before my baby was half a year old, I seldom went out, but after returning to work, I had more chances to go outside with my colleagues to get in touch with nature, which made me feel much more relaxed. In addition, I enjoy doing exercises that facilitate postpartum recovery, such as yoga.”[29].

“I spend some time at first adjusting to the changes in some work routines. Then I learned new things. During this time, there may be some new instruments, work routines… you have to learn and adapt to the change.”[27].

“My parents are now here to help me take care of the baby’s daily needs. Now I have more free time, and I don’t have as much work stress as before.”[29].

## 4. Discussion

This systematic review and qualitative meta‐synthesis draws on evidence from 19 studies to provide a comprehensive understanding of the clinical work experiences of nurses returning to practice postpartum. The analysis reveals this return period to be a time of profound multidimensional strain, characterized by the challenge of navigating intense dual‐role demands as a new mother and a clinician. A key finding is the critical role of a multilayered support ecosystem—encompassing interpersonal, organizational, and personal resources—which acts as a vital facilitator in navigating these challenges and promoting a successful return to work. By moving beyond broad narratives of work–family conflict to delineate the specific lived realities of this population, this review provides a nuanced evidence base that can inform targeted retention strategies for this experienced cohort within the global nursing workforce.

The core challenge elucidated is the navigation of dual demands, which manifests not merely as a logistical conflict but as a profound negotiation of identity. A pivotal finding is the breast feeding–work conflict, where the biological needs of lactation often conflict with the rigid spatiotemporal structures of clinical settings. The reported use of inadequate facilities for milk expression aligns with broader literature on occupational barriers to breastfeeding [40, 41], yet is acutely heightened in nursing due to unpredictable patient loads and often insufficient protected break times. This conflict transcends practicality, contributing to psychological distress and, for some, the premature cessation of breastfeeding [42]—a tangible health outcome adversely affected by workplace conditions. Simultaneously, the theme of reconciling professional and maternal identities reveals a spectrum of experiences. While role conflict and guilt are prevalent, the transformative potential of motherhood to enrich professional empathy and practice, particularly in specialties such as neonatal care, constitutes a significant and under‐acknowledged finding [14, 43]. This suggests that the postpartum period can serve as a catalyst for professional growth, a possibility often obscured by systemic challenges.

The physiological and psychological toll of this return period is captured in the second theme, enduring multidimensional strain. The pervasive physical depletion, compounded by sleep disruption and ongoing postpartum recovery, can place returning nurses at heightened risk for fatigue‐related errors and musculoskeletal injury. This aligns with studies on nurse fatigue [44–46] but is uniquely intensified by the concurrent demands of newborn care. Furthermore, professional return anxiety—specifically the fear of skill decay and a sense of being “de‐professionalized”—adds a critical dimension. This finding challenges assumptions that experienced nurses resume work seamlessly; instead, it highlights a vulnerable period of regaining competency that standard reorientation programs may not adequately address.

The most salient finding for intervention is the third theme, relying on a multilayered support ecosystem. The synthesis suggests that a successful return is heavily reliant upon support at multiple, interdependent levels. Interpersonal support, particularly from empathetic managers and peers, acts as an immediate buffer, consistent with job demands‐resources theory, wherein social support mitigates high job demands [47]. However, this review advances the literature by demonstrating that interpersonal goodwill is insufficient without organizational and structural enablers. The universal call for flexible work arrangements and adequate facilities points to a systemic policy‐practice gap. Our findings align with and extend calls for mandated lactation infrastructure and tailored return‐to‐work pathways [14, 24]. The active personal agency employed by nurses, while commendable, often functions to compensate for systemic failures rather than being optimally leveraged within a genuinely supportive framework.

### 4.1. Limitations

Several limitations should be considered. First, as a qualitative synthesis, it provides in‐depth insights but cannot establish the prevalence of these experiences across the broader nursing population. Second, the transferability of findings may be limited by an over‐representation of recent East Asian studies—which influences cultural themes like familial support—and by significant global disparities in maternity leave, lactation support, and childcare policies. Third, the review may be subject to methodological biases, including language bias (restriction to English and Chinese), search strategy bias (exclusion of gray literature by focusing on major databases), and publication bias (favoring significant or novel findings). Fourth, the synthesis likely omits the voices of nurses who left the profession postpartum, potentially skewing results toward those who persisted. Finally, the methodological quality of the included studies varied.

### 4.2. Implications for Nursing Practice and Research

To facilitate a sustainable return for postpartum nurses, healthcare organizations and leaders are encouraged to move beyond generic support to implement specific, actionable strategies. Based on our synthesis, we propose the following multilevel recommendations:

• Organizational Policy: Institutions should formalize phased return‐to‐work programs (e.g., gradual increase in full‐time equivalent) to mitigate physical exhaustion. Lactation support should ideally exceed minimum legal compliance by providing hospital‐grade pumps, protected pumping times that do not inadvertently penalize colleagues, and private, nonbathroom spaces. Additionally, exploring on‐site or subsidized childcare partnerships can alleviate the critical stressor of separation anxiety.
• Managerial and Educational Practice: Nurse managers should conduct structured pre‐return consultations to codesign schedules and discuss specific needs. To help address “professional return anxiety,” educators could offer refresher courses or simulation sessions on updated protocols. Implementing a peer mentorship or “buddy system” with experienced nurse‐mothers can provide essential emotional validation and practical navigation tips.
• Future Research: Future inquiry should move from descriptive studies to interventional research. Longitudinal, mixed‐methods designs are needed to quantify the impact of specific interventions (e.g., phased returns) on retention rates and long‐term maternal health outcomes. Furthermore, comparative studies across different healthcare systems could isolate the impact of specific policy variables on return success.

## 5. Conclusion

This meta‐synthesis provides a robust, evidence‐based framework for understanding the pivotal return period nurses undergo when returning to work postpartum. It highlights that their retention is not merely an individual concern but a systemic issue, closely tied to the presence of a coherent support ecosystem. Ultimately, supporting the sustainable reintegration of postpartum nurses is an essential investment. By prioritizing structural enablers and fostering a culture of psychological safety, healthcare systems can better retain experienced clinical expertise, thereby strengthening the resilience and quality of the global nursing workforce.

## Author Contributions

Jing Tian: topic formulation, conceptualization, design, methodology, writing–original draft, and writing–review and editing. Yuan Hu: validation and methodology. Yuting Yuan: data curation. Han Yang: validation, methodology, and data curation. Min Tan: validation, software, and data curation. Hong Zheng: validation, supervision, project administration, and writing–review and editing.

## Funding

The authors have nothing to report.

## Ethics Statement

As a systematic review and meta‐synthesis, this study did not involve primary data collection from human participants and therefore did not require additional ethical approval. The study was conducted in accordance with established principles of academic integrity and ethical research practice. All materials were derived from previously published studies that had secured the necessary ethical approvals prior to their implementation. We have made every effort to synthesize and present the findings of the original studies in a faithful and balanced manner, maintaining respect for their respective contexts and contributions.

## Conflicts of Interest

The authors declare no conflicts of interest.

## Supporting Information

Supporting A. The full search strategy is provided in supporting A.

Supporting B and supporting C. Furthermore, to ensure transparent reporting, this review adhered to the Preferred Reporting Items for Systematic Reviews and Meta‐Analyses (PRISMA) statement and the ENTREQ guidelines. Detailed checklists are provided in supporting B and supporting C.

Supporting D. The GRADE CERQual approach was used to assess the confidence in each included study finding: all subthemes were rated as high confidence. For details, see supporting D.

## Supporting information


**Supporting Information** Additional supporting information can be found online in the Supporting Information section.

## Data Availability

The authors have nothing to report.
