# Supplementary material for: Clinical Work Experiences of Nurses Returning to Work Postpartum: A Systematic Review and Qualitative Meta‐Synthesis
Source: J Nurs Manag. 2026 Apr 20;2026:8747372. doi: 10.1155/jonm/8747372 (PMC13095848; doi:10.1155/jonm/8747372)
Supplement: Supplementary file 1 — Supporting Information Additional supporting information can be found online in the Supporting Information section. [file JONM-2026-8747372-s001.zip › supplementary A ,Search strategy and results.docx]

**Supplementary A**

**Search strategy and results**

**Search Strategy and Results**

**Number of citations by each database register searched**

| **Databases** | **Citations** |
| --- | --- |
| Web of Science | 260 |
| Embase | 105 |
| PubMed | 892 |
| CINAHL | 115 |
| Cochrane | 144 |
| China Biomedical Literature Database(CBM) | 122 |
| Wanfang Database | 106 |
| Weipu Database | 21 |
| China National Knowledge Infrastructure(CNKI) | 44 |
| Other sources | 0 |
| **Total (databases)** | ****1809**** |

**Full search strategy for each database**

**PubMed**

# l:"nurs*" [Title/Abstract]

#2: ("Return to Work"[Mesh] OR "return to work"[tiab] OR "back to work"[tiab] OR "work resumption"[tiab] OR "Maternity Leave"[Mesh] OR "return*"[tiab] OR "postpartum return"[tiab] OR "parental leave")

#3:"experience*"  [Title/Abstract] OR" feeling*" [Title/Abstract]OR"interview*" [Title/Abstract]OR"attitude*"[Title/Abstract]OR"view*" [Title/Abstract]OR"perception"[Title/Abstract]OR"perspective*"[Title/Abstract]

#4:"qualitative research" [MeSH Terms] OR"qualitative study" [Title/ Abstract]OR "qualitative" [Title/Abstract]OR "content analysis" [Title/Abstract]OR"discourse analysis" [Title/Abstract]OR "ethnography" [Title/Abstract]OR "focus group" [Title/Abstract]OR "grounded theory"[Title/Abstract] OR "narrative" [Title/Abstract ]OR"phenomenology" [Title/Abstract]OR "thematic analysis" [Title/Abstract]

#5:#1 AND #2 AND #3AND #4

## Web of science

#1 TS=(nurs*)

#2 TS=(“return to work” OR “postpartum return” OR “back to work” OR “maternity leave” OR “parental leave”)

#3 TS=(experience* OR feeling* OR interview* OR attitude* OR perception OR view* OR perspective*)

#4 TS=("qualitative research" OR qualitative study* OR qualitative* OR content analysis* OR discourse analysis* OR ethnography* OR focus group* OR grounded theory* OR narrative* OR phenomenology* OR thematic analysis*)

#5 #1 AND #2 AND #3AND #4

**Cochrane**

((nurs* OR (nurse NEXT practitioner*) OR "nursing staff")):ti,ab,kw AND

(("return to work" OR "back to work" OR "work resumption" OR "maternity leave" OR "parental leave" OR (return* NEAR/3 work) OR postpartum)):ti,ab,kw AND

((experience* OR perception* OR perspective* OR view* OR attitude* OR feeling* OR "lived experience" OR qualitative)):ti,ab,kw AND

((qualitative OR (focus NEXT group*) OR interview* OR phenomenolog* OR "grounded theory" OR "thematic analysis" OR "content analysis")):ti,ab,kw

## **Embase**

## #1 'nurse'/exp OR nurs*:ti,ab,kw

## #2 'maternity leave'/exp OR 'parental leave'/exp OR 'return to work'/exp OR 'postpartum return':ti,ab,kw OR 'back to work':ti,ab,kw

## #3 experience*:ti,ab,kw OR feeling*:ti,ab,kw OR interview*:ti,ab,kw OR attitude*:ti,ab,kw OR perception:ti,ab,kw OR view*:ti,ab,kw OR perspective*:ti,ab,kw

## #4 'qualitative research'/exp OR 'qualitative research':ti,ab,kw OR 'qualitative study*':ti,ab,kw OR qualitative*:ti,ab,kw OR 'content analysis*':ti,ab,kw OR 'discourse analysis*':ti,ab,kw OR ethnography*:ti,ab,kw OR 'focus group*':ti,ab,kw OR 'grounded theory*':ti,ab,kw OR narrative*:ti,ab,kw OR phenomenology*:ti,ab,kw OR 'thematic analysis*':ti,ab,kw

## #5 #1 AND #2 AND #3 AND #4

## CINAHL

#1 (MH “Nurses+”) OR TI nurs* OR AB nurs*

#2 (MH “Maternity Leave+”) OR (MH “Return to Work+”) OR TI (“return to work” OR “postpartum return” OR “back to work” OR “parental leave”) OR AB (“return to work” OR “postpartum return” OR “back to work” OR “parental leave”)

#3 (experience* OR feeling* OR interview* OR attitude* OR perception OR view* OR perspective*) OR AB (experience* OR feeling* OR interview* OR attitude* OR perception OR view* OR perspective*)

#4 (MH “Qualitative Studies+”) OR TI (“qualitative research” OR “qualitative study*” OR qualitative* OR “content analysis*” OR “discourse analysis*” OR ethnography* OR “focus group*” OR “grounded theory*” OR narrative* OR phenomenology* OR “thematic analysis*”) OR AB (“qualitative research” OR “qualitative study*” OR qualitative* OR “content analysis*” OR “discourse analysis*” OR ethnography* OR “focus group*” OR “grounded theory*” OR narrative* OR phenomenology* OR “thematic analysis*”)

#5 S1 AND S2 AND S3 AND S4

**China Biomedical Literature Database**

( ("护士"[常用字段:智能] OR "护理人员"[常用字段:智能] OR "助产士"[常用字段:智能] ) AND ( "返岗"[常用字段:智能] OR "重返工作"[常用字段:智能] OR "复工"[常用字段:智能] OR "产后返岗"[常用字段:智能] OR "重返工作岗位"[常用字段:智能] OR "重返职场"[常用字段:智能] OR "产假"[常用字段:智能] ) AND ( "体验"[常用字段:智能] OR "感受"[常用字段:智能] OR "访谈"[常用字段:智能] OR "态度"[常用字段:智能] OR "看法"[常用字段:智能] OR "认知"[常用字段:智能] OR "视角"[常用字段:智能] OR "观点"[常用字段:智能] OR "心理"[常用字段:智能] OR "质性研究"[常用字段:智能] OR "定性研究"[常用字段:智能] OR "现象学"[常用字段:智能] OR "扎根理论"[常用字段:智能] OR "叙事"[常用字段:智能] OR "内容分析"[常用字段:智能] OR "主题分析"[常用字段:智能] ) )

**WANFANG**

主题:("护士" or "护理人员" or "助产士" or "注册护士" or "临床护士") and 主题:("返岗" or "重返工作" or "复工" or "产后返岗" or "重返工作岗位" or "重返职场" or "产假后") and 主题:("体验" or "感受" or "访谈" or "态度" or "看法" or "认知" or "视角" or "观点" or "心理" or "质性研究" or "定性研究" or "现象学" or "扎根理论" or "叙事研究" or "内容分析" or "主题分析")

**Weipu**

(M=护士 OR M=护理人员 OR M=助产士) AND (M=返岗 OR M=重返工作 OR M=复工 OR M=产后返岗 OR M=重返工作岗位 OR M=重返职场 OR M=产假后) AND (M=体验 OR M=感受 OR M=访谈 OR M=态度 OR M=看法 OR M=认知 OR M=视角 OR M=观点 OR M=质性研究 OR M=定性研究 OR M=现象学 OR M=扎根理论 OR M=叙事研究 OR M=内容分析 OR M=主题分析)

**China National Knowledge Infrastructure**

(SU='护士' OR SU='护理人员' OR SU='助产士' OR SU='注册护士') AND (SU='返岗' OR SU='重返工作' OR SU='复工' OR SU='产后返岗' OR SU='产假后返岗' OR SU='重返工作岗位' OR SU='产后复工' OR SU='重返职场') AND (SU='体验' OR SU='感受' OR SU='访谈' OR SU='态度' OR SU='看法' OR SU='认知' OR SU='视角' OR SU='观点' OR SU='心理' OR SU='质性' OR SU='质性研究' OR SU='现象学' OR SU='扎根理论' OR SU='叙事研究' OR SU='内容分析' OR SU='主题分析' OR SU='Meta整合')
