# Supplementary material for: Clinical Work Experiences of Nurses Returning to Work Postpartum: A Systematic Review and Qualitative Meta‐Synthesis
Source: J Nurs Manag. 2026 Apr 20;2026:8747372. doi: 10.1155/jonm/8747372 (PMC13095848; doi:10.1155/jonm/8747372)
Supplement: Supplementary file 1 — Supporting Information Additional supporting information can be found online in the Supporting Information section. [file JONM-2026-8747372-s001.zip › supplementary D GRADE-CERQual evidence profile.docx]

**Supplementary D GRADE-CERQual evidence profile.**

| **Summary of review finding** (i.e., subtheme) | **Studies contributing to the review finding** (first author, year,country) | **Assessment of methodological limitations** | **Assessment of relevance** | **Assessment of coherence** | **Assessment of adequacy** | **Overall CERQual assessment of confidence** | **Explanation of**  **judgement** |
| --- | --- | --- | --- | --- | --- | --- | --- |
| **Theme 1. The Multi-Faceted Challenges of Reintegrating into Professional Practice** | | | | | | |  |
| **Subtheme 1.1. Physiological and Infrastructural Barriers** | Hill et al., 2023, USA; Riaz & Condon, 2019, Pakistan;Li, 2024, China;Chen et al., 2024, China;Cheng et al.2025,China;Xie et al.2026,China | ****Minor limitations**** | ****Highly relevant**** | ****Coherent**** | ****Rich data**** | ****High**** | All six studies employed appropriate qualitative designs (descriptive, phenomenological). The findings consistently highlight core barriers: physical challenges (fatigue, pain, sleep deprivation) and workplace infrastructure deficits (lack of lactation rooms, rigid policies). Data from 84 participants across the USA, Pakistan, and China provide thick description and thematic saturation. |
| **Subtheme 1.2. Reconciling Professional and Maternal Identities** | Chen et al., 2024, China; Liu et al., 2024, China; Zhou et al., 2024, China;Li et al., 2025, China;Costantini et al., 2022, Italy;Huang etal.2018,China;Yang et al.2024,China | ****Minor limitations**** | ****Directly relevant**** | ****Consistent across studies**** | ****Rich, well-saturated data**** | ****High**** | Seven qualitative studies from China, Italy, and Taiwan used rigorous phenomenological approaches. All directly explore identity negotiation, consistently reporting role confusion, job-ability mismatch, and dual burden of professional and maternal expectations. Data from 107 participants are rich, with detailed illustrative quotes and clear thematic convergence. |
| **Subtheme 1.3. Maternal Separation and Childcare Logistics** | Liu et al., 2024, China;Li, 2024, China; Costantini et al., 2022, Italy; Zhou et al., 2024, China;Tseng et al., 2023, Taiwan; Riaz & Condon, 2019, Pakistan;Guo et al.2024,China;He et al.2020,China;Xiang et al.2023,China | ****Minor limitations**** | ****Highly relevant**** | ****Coherent**** | ****Rich, multi-context data**** | ****High**** | Nine studies across multiple regions (China, Italy, Taiwan, Pakistan) consistently identify separation anxiety and childcare arrangements as primary stressors. Methodologies are sound and appropriate. The dataset includes 125 participants, providing extensive, thick description and achieving saturation across all contexts. No conflicting findings. |
| **Theme 2. Enduring Multidimensional Physical and Psychological Strain** | | | | | | |  |
| **Subtheme 2.1. Physical Depletion and Health Concerns** | Chen et al., 2024, China; Liu et al., 2024, China;Li, 2024, China;Li et al.2017,China;Xie et al.2026,China | ****Minor limitations**** | ****Directly relevant**** | ****Coherent**** | ****Rich data**** | ****High**** | Six studies from China and All employed appropriate qualitative methods (phenomenology, descriptive design). Data from 77 participants show highly coherent themes of bodily exhaustion and health-related anxiety post-return. |
| **Subtheme 2.2. Emotional and Cognitive Burden** | Li, 2024, China; Khalil & Davies, 2000, UK;Zhou et al., 2024, China; Li et al., 2025, China;Liu et al., 2024, China; Cheng et al.2025,China;Li et al.2017,China | ****Minor to moderate limitations**** (one older study from 2000) | ****Highly relevant**** | ****Highly coherent**** | ****Very rich and well-saturated**** | ****High**** | Eight studies spanning 2000–2026 consistently report emotional burdens: guilt, anxiety, worry, irritability, fear, and maternal separation distress. Despite one older study, recent high-quality research strongly reinforces these findings. The dataset includes 97 participants with thick, convergent narratives across the UK, USA, and China. |
| **Subtheme 2.3. Professional Reintegration Anxiety** | Tseng et al., 2023, Taiwan; Costantini et al., 2022, Italy);Li, 2024, China;Liu et al., 2024, China;He et al.2020,China;Yang et al.2024,China | ****Minor limitations**** | ****Directly relevant**** | ****Coherent**** | ****Adequate**** | ****Moderate**** | All six studies specifically explore anxiety related to skill loss, competency fears, and re-adaptation to clinical environments. Qualitative designs are robust. Findings are congruent across Asian and European contexts. Data from 82 participants are sufficient, with clear thematic saturation and illustrative quotes. |
| **Theme 3. Relying on a Multilayered Support Ecosystem for Successful Transition** | | | | | | |  |
| **Subtheme 3.1. Interpersonal Support as a Critical Buffer** | Riaz & Condon, 2019, Pakistan; Zhou et al., 2024, China;Wan et al., 2024, China; Huang etal.2018,China;Xie et al.2026,China | ****Minor limitations**** | ****Highly relevant**** | ****Consistent across settings**** | ****Rich data**** | ****High**** | Five studies from Pakistan, China, and the USA uniformly highlight the importance of family, partner, and peer support. Methodologies are sound (thematic analysis, phenomenological approaches). Data from 66 participants provide thick description and well-saturated themes on support mechanisms. |
| **Subtheme 3.2. Organizational and Structural Enablers** | Zhou et al., 2024, China;Hill et al., 2023, USA;Riaz & Condon, 2019, Pakistan; Liu et al., 2024, China;He et al.2020,China;Xiang et al.2023,China | ****Minor limitations**** | ****Directly relevant**** | ****Coherent**** | ****Adequate**** | ****Moderate**** | Six studies address institutional factors: flexible scheduling, lactation facilities, managerial support, and policy gaps. Designs are appropriate. Findings are consistent across cultures (USA, Pakistan, China). Data from 86 participants are credible and thematically saturated. |
| **Subtheme 3.3. Personal Agency and Coping Strategies** | Chen et al., 2024, China; Zhou et al., 2024, China;Li, 2024, China; Tseng et al., 2023, Taiwan;Wan et al., 2024, China;Cheng et al.2025,China;Huang etal.2018,China | ****Minor limitations**** | ****Highly relevant**** | ****Coherent**** | ****Rich, multi-context data**** | ****High**** | Seven qualitative studies from China and Taiwan explore active coping strategies: self-adjustment, communication, skill renewal, and positive self-management. Methodologies are rigorous. Themes are convergent. Data from 99 participants are rich and well-supported by detailed narratives. |
